# Clofibrate inhibits the umami-savory taste of glutamate

**DOI:** 10.1371/journal.pone.0172534

**Published:** 2017-03-01

**Authors:** Matthew Kochem, Paul A. S. Breslin

**Affiliations:** 1 Rutgers University Department of Nutritional Sciences, New Brunswick, NJ, United States of America; 2 Monell Chemical Senses Center Philadelphia, PA, United States of America; Duke University, UNITED STATES

## Abstract

In humans, umami taste can increase the palatability of foods rich in the amino acids glutamate and aspartate and the 5’-ribonucleotides IMP and GMP. Umami taste is transduced, in part, by T1R1-T1R3, a heteromeric G-protein coupled receptor. Umami perception is inhibited by sodium lactisole, which binds to the T1R3 subunit *in vitro*. Lactisole is structurally similar to the fibrate drugs. Clofibric acid, a lipid lowering drug, also binds the T1R3 subunit *in vitro*. The purpose of this study was to determine whether clofibric acid inhibits the umami taste of glutamate in human subjects. Ten participants rated the umami taste intensity elicited by 20 mM monosodium glutamate (MSG) mixed with varying concentrations of clofibric acid (0 to 16 mM). In addition, fourteen participants rated the effect of 1.4 mM clofibric acid on umami enhancement by 5’ ribonucleotides. Participants were instructed to rate perceived intensity using a general Labeled Magnitude Scale (gLMS). Each participant was tested in triplicate. Clofibric acid inhibited umami taste intensity from 20 mM MSG in a dose dependent manner. Whereas MSG neat elicited “moderate” umami taste intensity, the addition of 16 mM clofibric acid elicited only “weak” umami intensity on average, and in some subjects no umami taste was elicited. We further show that 1.4 mM clofibric acid suppressed umami enhancement from GMP, but not from IMP. This study provides *in vivo* evidence that clofibric acid inhibits glutamate taste perception, presumably via T1R1-T1R3 inhibition, and lends further evidence that the T1R1-T1R3 receptor is the principal umami receptor in humans. T1R receptors are expressed extra-orally throughout the alimentary tract and in regulatory organs and are known to influence glucose and lipid metabolism. Whether clofibric acid as a lipid-lowering drug affects human metabolism, in part, through T1R inhibition warrants further examination.

## Introduction

Human umami (or savory) taste perception is typically elicited by select amino acids, such as glutamate and aspartate, and certain 5’-ribonucleotides, such as inosine and guanosine. Umami taste is hypothesized to have evolved to guide the ingestion of foods rich in these compounds, including certain vegetables and meats, as well as any fermented, aged, or cooked foods [1]. Recently, umami and sweet taste receptors have been implicated as regulators of metabolic physiology as well [2].

*In vitro* functional expression data and mouse gene knock-out studies suggest that glutamate taste perception is transduced, in part, by the heteromeric G-protein coupled receptor (GPCR) T1r1-T1r3 and possibly also by shortened splice variants of mGluR1 and mGluR4 receptors [3,4,5,6,7], as well as a variant of the N-methyl-D-Aspartate (NMDA) receptor [8,9]. Heterologously expressed human T1R1-T1R3 is activated *in vitro* by L-glutamate and is enhanced by the 5’ ribonucleotides inosine monophosphate (IMP) and guanosine monophosphate (GMP) [6,10]. 5’ ribonucleotides are thought to bind a site near the T1R1 venus flytrap domain, thereby stabilizing the closed, activated conformation [11].

T1R3-KO mice exhibit greatly reduced, but not abolished, preference for MSG at concentrations between 30 and 300 mM [12]. T1R3 is essential for chorda tympani (CT) nerve responses to MSG and IMP. T1R3 does not, however, play a necessary role in glossopharyngeal (GL) nerve responses to either MSG alone or MSG+IMP. This suggests that other receptors mediate GL responses to glutamate and ribonucleotides [12]. Similar effects were observed in T1R1-KO mice [13]. Ablation of T1R1 reduces but does not abolish neural and behavioral responses to glutamate plus IMP. And, T1R1-KO does not affect GL nerve responses to glutamate. Additionally, mGluR antagonists further reduce neural and behavioral responses to amino acid stimuli, suggesting a role for mGluRs in glutamate taste [13]. Taken together, these studies suggest that T1R1-T1R3, mGluRs, and possibly other receptors may all be involved in glutamate taste responses.

Human umami perception from L-glutamate is inhibited by sodium lactisole, which has been shown *in vitro* to bind to the T1R3 transmembrane domain [3,14,15]. Although lactisole is a more potent inhibitor of sweet taste than umami taste, it has been shown to increase detection thresholds for glutamate by four fold in human subjects [3,15]. Lactisole is structurally similar to other phenyl propionic acids including the fibrate drugs, a class of lipid lowering pharmaceuticals [16]. Clofibric acid reduces plasma lipid levels, improves glucose tolerance, and reduces ectopic lipid deposition. Similar to lactisole, clofibric acid binds the transmembrane domain of T1R3 and inhibits T1R1-T1R3 activity *in vitro* [16]. Although clofibric acid is known to impart effects via PPARα agonism, it is not known whether its physiological effects may also be due, in part, to T1R3 inhibition.

There is mounting evidence that stimulation of extra-orally expressed T1R3 influences metabolism. T1R3 is not only expressed in the oral cavity, but also in the intestine, pancreas, liver, adipose, cardiac and skeletal muscle, and hypothalamus [17,18,19]. Stimulation of T1Rs on enteroendocrine cells triggers incretin release (such as glucagon like peptide-1 [GLP-1]), which promote luminal glucose transport and stimulate insulin secretion [20,21]. Stimulation of T1r1-T1r3 in intestinal L-cells stimulates cholecystokinin (CCK) release [22], which promotes bile secretion and satiation. Non-nutritive sweeteners, which bind T1R2-T1R3, affect glycemic and hormonal responses to glucose consumption [23,24,25,26]. Lactisole, a structural homolog of clofibric acid, has been shown to affect blood glucose and hormone responses when ingested [16,27]. *T1r3* knockout animals fed obesogenic diets have reduced adiposity and smaller adipocytes relative to wildtypes [28].

It is a reasonable hypothesis, therefore, that some of clofibric acid’s physiological effects could be mediated by inhibition of the T1R3 subunit in the carbohydrate receptor T1R2-T1R3 and the amino acid receptor T1R1-T1R3. In order to determine whether clofibric acid imparts T1R3-mediated effects on human health, we must first verify that clofibric acid inhibits T1R3 functions *in vivo*. Umami taste perception presents a convenient means of studying T1R3 function in humans *in vivo*. The purpose of this study was to determine whether clofibric acid inhibits the perception of umami taste in humans and is, thus, an amino acid taste receptor inhibitor *in vivo*.

## Materials and methods

### Subjects

24 adult subjects were paid to participate after providing their informed consent on Rutgers University Institutional Review Board (IRB) approved forms. All participants were from Rutgers University and the surrounding community. Participants were asked not to eat, drink, or smoke one hour prior to each session. This protocol complies with the Declaration of Helsinki for Medical Research involving human subjects and the study was approved by the Institutional Review Board at Rutgers University.

### Training

Subjects were trained in the use of a general Labeled Magnitude Scale (gLMS) following standard published procedures [29]. The top of the scale was described as the strongest imaginable sensation of any kind. Participants were instructed to rate perceived intensity along a vertical axis lined with the following adjectives: barely detectable, weak, moderate, strong, very strong, and strongest imaginable. The adjectives were spaced semi-logarithmically, based upon experimentally determined intervals to yield ratio quality data.

### Effect of clofibric acid on 20 mM MSG

Ten participants were asked to evaluate umami intensity from monosodium glutamate (MSG) mixed with clofibric acid (CF). MSG concentration was fixed at 20 mM, as this was the level used by Galindo-Cuspinera that was inhibited effectively by lactisole [14]. 20 mM MSG was mixed with varying concentrations of clofibric acid to examine its inhibitory function. The concentrations of clofibric acid used were 0, 1, 4, 8, and 16 mM. Clofibric acid was neutralized with sodium hydroxide to match the pH of the neat MSG solution.

### Effect of 1.37 mM clofibric acid on MPG and ribonucleotides

Fourteen participants were asked to evaluate the effect of clofibric acid on umami taste enhancement by 5’ ribonucleotides. The compounds used were monopotassium glutamate (MPG), inosine monophosphate disodium salt (IMP), guanosine monophosphate disodium salt (GMP), and clofibric acid (CF). MPG was used instead of MSG so that umami taste, rather than salty taste, was predominant. MPG was prepared in a range of concentrations (3.16 to 316 mM) increasing in quarter logarithmic increments. The MPG series was prepared in six conditions: neat, with 1.4 mM clofibric acid, with 3 mM IMP, with 3 mM GMP, with IMP + clofibric acid, and with GMP + clofibric acid. This dose of clofibric acid has been shown to inhibit sweetness intensity from high potency sweeteners and sucrose [30]. Two participants were excluded from the final analysis on the basis that they reported no difference in umami intensity between 3.16 and 316 mM MPG, indicating they were insensitive to changes in glutamate concentration over two orders of magnitude. Exclusion of these participants did not change the outcomes of the statistical analyses.

### Stimulus delivery

Aqueous solutions were prepared every other day with Millipore filtered water and stored in amber glass at 4°C. All solutions were brought to room temperature one hour prior to tasting. All solutions were fully dissolved and there were no visible signs of undissolved solids or precipitation from solutions. Millipore_TM_ filtered water was used as the solvent and rinsing agent in all experiments.

Sample presentation was randomized using a random integer generator (random.org) and 10 mls of each solution was presented in 30 ml polyethylene medicine cups (Dynarex, Orangeburg, NY) on a tray with numbered cup spaces. Each series was tested in triplicate with an inter-stimulus interval of 75 s and a five-minute interval between trials. For each sample, subjects held 10 ml of solution in the mouth for 5 s and rated the taste qualities (sweet, bitter, salty, sour, savory) and intensity before expectorating.

### Statistical analysis

Data were log transformed for analyses and presented as geometric means +/- geometric standard error.

To analyze inhibition of glutamate taste by clofibric acid, one-way repeated measures analysis of variance was conducted with clofibric acid concentration as factor. Pairwise Tukey honestly significant difference (HSD) tests were used to find significant differences in umami intensity ratings between clofibric acid concentrations.

To analyze enhancement of umami intensity ratings from 5’ ribonucleotides, 2-ways repeated measures ANOVA was conducted with glutamate concentration and stimulus (MPG neat, MPG+IMP, MPG+GMP) as factors. To analyze inhibition of glutamate + ribonucleotides from clofibric acid, 2-ways repeated measures ANOVA was conducted with glutamate concentration and stimulus (MPG+IMP, MPG+IMP+CF, MPG+GMP, MPG+GMP+CF) as factors. Pairwise Tukey HSD tests were used to find significant differences between stimuli. Alpha was set at p<0.05.

## Results

### Inhibition of glutamate taste by clofibric acid

**Fig 1:**
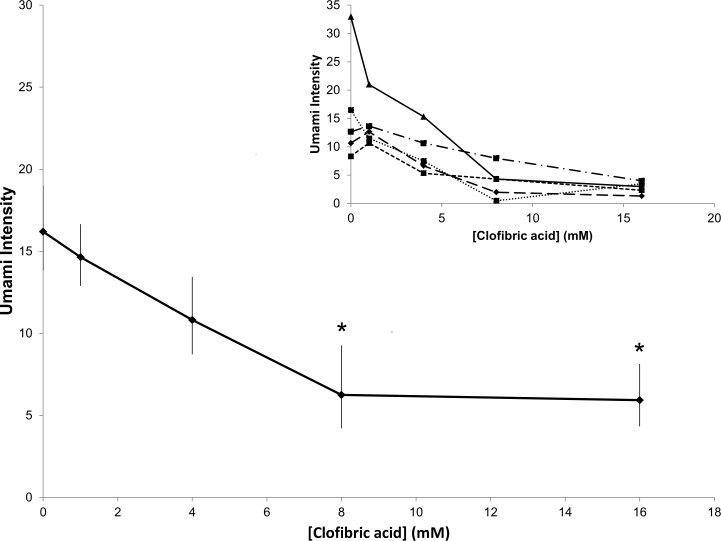
Clofibric acid inhibits umami intensity in a dose dependent manner. 8 mM and 16 mM clofibric acid significantly reduced umami intensity from MSG. Umami perception was not abolished on average by any concentration of clofibric acid. Data are geometric means +/- standard error. Data were collected from 10 participants, each tested in triplicate. * indicates significant difference from 0 mM clofibric acid at α = 0.05. (Inset) Data from 5 individuals showing full umami inhibition from clofibric acid.

Clofibric acid inhibited the umami taste of 20 mM MSG in a dose dependent manner (**Fig 1**). One-way repeated measures ANOVA showed a significant effect of clofibric acid concentration on perceived umami intensity (F[4, 36] = 6.979, p<0.001). Posthoc tests showed that 8 and 16 mM clofibric acid significantly reduced umami intensity relative to 0 mM clofibric acid (p<0.01) and 1 mM clofibric acid (p<0.05). Umami taste intensity was reduced by approximately 60% from moderate to weak. In five participants, 8 and 16 mM clofibric acid abolished umami intensity (Fig 1 inset).

### Interactions of glutamate, 5’ ribonucleotides, and clofibric acid

**Fig 2:**
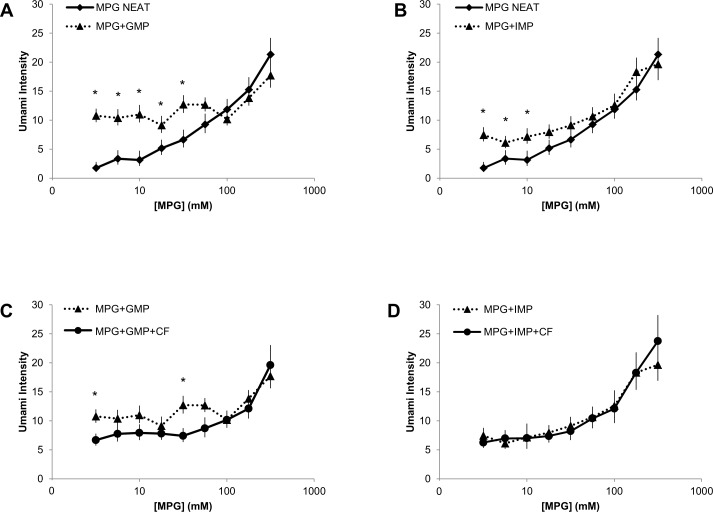
Clofibric acid differentially inhibits umami enhancement from 5’ ribonucleotides. (**A, B**) Effect of 5’ ribonucleotides on umami taste intensity from an MPG concentration series. MPG was prepared across a broad range of concentrations and presented neat, with 3 mM GMP added, and with 3 mM IMP added. GMP and IMP enhanced umami intensity from MPG. (**C, D**) Effect of clofibric acid on umami taste enhancement from 5’ ribonucleotides. MPG was prepared across a broad range of concentrations and presented with 3 mM GMP, 3 mM IMP, GMP+ 1.4 mM CF, and IMP+ 1.4 mM CF. The addition of 1.4 mM clofibric acid inhibited umami intensity from MPG when prepared in mixture with GMP, but not with IMP. Data are geometric means +/- standard error. Data are presented from 12 participants, each tested in triplicate. * indicates significant difference from MPG neat (A,B) and MPG + GMP (C) at α = 0.05.

IMP and GMP significantly enhanced perceived umami intensity from MPG (Fig 2A and 2B). Two-ways repeated measures ANOVA showed a significant effect of 5’ ribonucleotide on umami intensity ratings (F[2, 22] = 7.15, p<0.01). There was an interaction of stimulus and concentration (F[16,176] = 7.15, p<0.001), which reflects the pronounced enhancement of umami intensity at low concentrations of MPG. Enhancement was slightly greater with GMP relative to IMP. Simple main effects analysis showed that MPG+GMP elicited stronger umami taste intensity than did MPG+IMP at 3.16 mM MPG (p<0.05) and 5.62 mM MPG (p<0.05), but there were no significant differences between the ribonucleotides at higher MPG concentrations. Clofibric acid inhibited umami intensity from MPG+GMP (Fig 2C), but not from MPG+IMP, (Fig 2D). Two-ways repeated measured ANOVA revealed an effect of stimulus (F[3,33] = 1.89, p = 0.15) and an interaction of stimulus and concentration (F[24,264] = 3.28, p<0.001). Simple main effects analysis showed that clofibric acid significantly reduced umami taste intensity from MPG+GMP, which post-hoc analysis revealed was significant at 3.16 mM MPG (p<0.01) and 31.6 MPG (p<0.01) (Fig 2C).

## Discussion

These data show that clofibric acid inhibits in a dose dependent manner the umami taste intensity of 20 mM glutamate (**Fig 1**). In addition, clofibric acid inhibited umami intensity from glutamate prepared with GMP, but not IMP (**Fig 2**). Collectively, these data suggest that clofibric acid, which binds T1R3 *in vitro*, is a potent umami taste inhibitor *in vivo*, presumably via inhibition of the T1R3 subunit of the T1R1-T1R3 receptor. An *in vitro* study of T1R2-T1R3 by Maillet and colleagues showed that both lactisole and clofibric acid bind the transmembrane domain of T1R3 [16]. Thus, it appears that both lactisole and clofibrate inhibit the umami taste from glutamate by binding with similar affinities to T1R1-T1R3. The observation that high concentrations of clofibric acid completely inhibited umami taste in five subjects (Fig 1 inset), suggests that T1R1-T1R3 is the predominant elicitor of umami taste in humans. Although we cannot rule out at this time the inhibition of other candidate umami taste receptors, mGluR1, mGluR4, or NMDA, by clofibric acid.

1.4 mM clofibric acid modestly inhibited umami intensity of a range of glutamate concentrations prepared in mixture with 3 mM GMP (**Fig 2C**), but not with 3 mM IMP (**Fig 2D**). Thus, clofibric acid and lactisole behave differently as inhibitors of enhanced umami taste perception, since we have previously reported that lactisole does not inhibit umami taste in mixture with GMP and IMP [14]. GMP and IMP bind the T1R1 subunit, and this binding stabilizes the closed, activated conformation of the receptor, thereby enhancing activation of the receptor by glutamate [11]. We hypothesized that ribonucleotide binding alters the conformation of T1R3 such that its affinity for lactisole is reduced [14]. We predicted that clofibric acid would not inhibit umami taste intensity when prepared in mixture with 5’ ribonucleotides. It is unclear why clofibric acid inhibited taste perception from stimulation with MPG +GMP but not MPG + IMP. But this observation indicates that GMP and IMP interact with the T1R3 slightly differently. The relative effects of 5’ ribonucleotides on umami taste perception should be investigated further. It is also possible that greater doses of clofibric acid could elicit more pronounced inhibition of umami taste from MPG and ribonucleotides. The clofibrate dose of 1.4 mM was selected based on our previous work that showed it inhibits sweetness intensity across broad concentration ranges of sweeteners [30]. The difference in inhibitory efficacy suggests that clofibric acid is a superior inhibitor of sweetness intensity than umami intensity. These observations could be tentatively explained by at least two mechanisms; first, that clofibric acid’s inhibitory effect may be altered by the identity of the T1R3 heteromer partner (T1R1 vs T1R2), and second, that umami perception is determined not only by T1Rs, but also mGluRs and perhaps other receptors. In the present study, however, clofibrate completely inhibited umami taste in five participants (Fig 1, inset), which suggests a dominant role of T1Rs in human umami perception.

There is mounting evidence that extra-orally expressed T1Rs influence metabolism. Possibly related to its T1R inhibitory activity, clofibric acid treatment is associated with reduction of fasting and postprandial glucose concentrations in human patients [31,32,33]. And treatment with clofibric acid may improve insulin sensitivity by reducing lipid content in skeletal muscle, liver, and other tissues [34]. Clofibric acid is known to reduce lipid stores in these tissues via the presumed mechanism of PPAR-α agonism [35]. PPAR-α is a transcription factor which upregulates genes responsible for fatty acid uptake, beta oxidation, lipolysis, and apolipoprotein synthesis [36]. Clofibric acid also down-regulates the synthesis of fatty acids and triglycerides.

Importantly, the amount of clofibric acid typically used in human patients to treat hyperlipidemia is also adequate to block T1R1-T1R3 and T1R2-T1R3 activity. The IC50 of clofibric acid for inhibiting T1R2-T1R3 is similar to the EC50 of clofibric acid for activating PPAR-α [16,37]. The amount of lactisole demonstrated to alter glucose tolerance acutely is 135 mg dissolved in 300 ml (2 mM) [27]. The typical dose of clofibric acid used to treat heart disease is 2000 mg/day, divided into 500 mg capsules administered throughout the day [38,39]. This level would be expected to inhibit T1R3 activity in the alimentary tract. Among our subjects, the IC50 for clofibric acid inhibition of umami taste was ~ 4 mM (**Fig 1**), which approximates the concentration of clofibric acid in the alimentary tract and plasma following consumption of 500 mg doses.

In the present study, 8 and 16 mM clofibric acid significantly reduced perceived umami intensity elicited by 20 mM MSG, comparable to the lactisole results of Galindo-Cuspinera & Breslin [14]. Clofibric acid also inhibited perceived umami intensity from MPG + GMP. This provides the first *in vivo* evidence that clofibric acid inhibits umami taste, likely acting via inhibition of T1R1-T1R3, as was demonstrated *in vitro*. Clofibric acid and other fibrate drugs inhibit T1R activity in the alimentary tract at levels that are typically ingested for cholesterol treatment. Whether the exposure to clofibrate and the resulting T1R inhibition alters metabolism and the handling of amino acids, carbohydrates, and lipids warrants future examination.

## Supporting information

S1 File Copy of BRESLIN data for PLOS ONE submission. (XLSX)
